# Transcriptomic profiling reveals autophagy-associated programs and prognostic biomarkers in anoikis-resistant pancreatic cancer cells

**DOI:** 10.1186/s12885-026-17107-7

**Published:** 2026-10-13

**Authors:** Lizhi Lin, Monika Bauden, Roland Andersson, Daniel Ansari

**Affiliations:** 1 Department of Breast Surgery, the First Affiliated Hospital of Wenzhou Medical University, Wenzhou, China; 2 Department of Surgery, Clinical Sciences Lund, Lund University, Skåne University Hospital, Lund, Sweden

**Keywords:** Pancreatic ductal adenocarcinoma, Anoikis resistance, Autophagy, Transcriptomic profiling, UBC, ATG14, Metastasis

## Abstract

Pancreatic ductal adenocarcinoma (PDAC) is characterized by aggressive metastatic progression and poor clinical outcomes. During metastatic dissemination, detached tumor cells must overcome anoikis, a form of apoptosis induced by loss of extracellular matrix attachment. Although anoikis resistance is considered a critical prerequisite for metastasis, the adaptive mechanisms supporting detached survival in pancreatic cancer remain incompletely understood. In this study, anoikis-resistant (AR) pancreatic cancer cell models were established through repeated long-term suspension culture using Capan-1 and PSN-1 cells. Transcriptomic profiling was performed to identify conserved molecular programs associated with anoikis resistance in PDAC. Gene set variation analysis (GSVA) demonstrated consistent transcriptomic enrichment of autophagy-associated and suppression of cell cycle-related pathways in both AR models. Differential expression and integrative pathway analyses further identified several core genes associated with autophagy regulation and cytokine signaling. Survival analysis revealed that elevated expression of UBC and ATG14 was associated with poor prognosis in pancreatic cancer patients. Our findings suggest that suspension-selected pancreatic cancer cells undergo coordinated stress-adaptive transcriptional remodeling characterized by enriched autophagy- and ubiquitin-associated transcriptional programs.

## Introduction

Pancreatic ductal adenocarcinoma (PDAC) remains one of the most lethal malignancies worldwide, with a 5-year survival rate below 15% [1]. The poor prognosis of PDAC is largely attributed to its aggressive metastatic behavior and the fact that most patients present with advanced disease at diagnosis [2, 3]. Increasing evidence suggests that pancreatic cancer dissemination can occur early during tumor evolution, allowing tumor cells to enter the circulation and colonize distant organs such as the liver and lung [4, 5]. To survive systemic dissemination, detached tumor cells must overcome anoikis, a specialized form of apoptosis induced by loss of extracellular matrix (ECM) attachment [6, 7]. Anoikis resistance is therefore considered a critical prerequisite for metastatic progression [8, 9]. Anoikis resistance is a hallmark of metastatic cancer cells, enabling them to survive detachment from the extracellular matrix and disseminate through circulation [10]. Pancreatic cancer cells exhibit a high metastatic potential and poor prognosis, yet the molecular mechanisms underlying anoikis resistance remain not fully elucidated.

Following detachment from the extracellular matrix, tumor cells experience cellular stress caused by disruption of integrin signaling, metabolic imbalance, oxidative stress, and impaired nutrient acquisition [7, 11, 12]. These conditions can rapidly trigger anoikis and other forms of stress-induced cell death. To survive during dissemination, detached cancer cells must therefore undergo adaptive transcriptional and metabolic reprogramming that enables maintenance of energy homeostasis, proteostasis, and redox balance [13, 14]. Increasing evidence suggests that anoikis resistance is not mediated by a single signaling pathway but rather reflects a coordinated stress-adaptive state involving multiple survival mechanisms [15].

Recent studies have further highlighted extensive crosstalk between the ubiquitin-proteasome system and autophagy during cellular stress adaptation [16]. Ubiquitination regulates selective autophagy by labeling damaged proteins and organelles for degradation, thereby contributing to mitochondrial quality control and survival under stress conditions [16, 17]. Dysregulation of ubiquitin-associated signaling has been implicated in pancreatic cancer progression, metastasis, and therapeutic resistance [18]. Nevertheless, the molecular relationship between ubiquitin-associated regulation, autophagy activation, and anoikis resistance in PDAC remains poorly defined.

In the present study, we generated anoikis-resistant derivates of the human pancreatic cancer cell lines PSN-1 and Capan-1, originating from primary tumor or metastatic lesion, respectively. Transcriptomic profiling was subsequently performed to identify conserved adaptive programs associated with anoikis resistance. Integrative pathway and differential expression analyses revealed coordinated transcriptomic enrichment of autophagy- and ubiquitin-associated pathways together with suppression of cell-cycle-related programs. Further survival analysis identified several core genes associated with patient prognosis, including UBC and ATG14. Our findings suggest that ubiquitin- and autophagy- associated transcriptional programs may be associated with the suspension-selected phenotype of pancreatic cancer cell under detachment stress.

## Materials and methods

### Cell culture

Human pancreatic cancer cell lines PSN-1 and Capan-1 were obtained from the American Type Culture Collection (ATCC, Manassas, VA, USA). PSN-1 cells were cultured in RPMI-1640 medium (Gibco, UK) supplemented with 10% fetal bovine serum (FBS; Gibco, UK) and 1% penicillin-streptomycin solution (100 U/mL penicillin and 100 μg/mL streptomycin; Gibco, USA). Capan-1 cells were cultured in IMDM medium (Gibco, UK) supplemented with 20% FBS and 1% penicillin-streptomycin. The cells were maintained at 37 °C in a humidified incubator with 5% CO₂.

### Anoikis-resistant cell line

Anoikis-resistant pancreatic cancer cell lines were established by repeated cycles of suspension culture in ultra-low attachment plates (Corning) followed by recovery in adherent condition. Surviving cells were collected and expanded after each cycle. Both PSN-1 and Capan-1 cells were subjected to 48 h suspension/re-attachment cycles. Sequential selection was continued for approximately 2–3 months until stable anoikis-resistant populations were obtained. Resistance to anoikis was verified by western blot analysis of apoptosis-related proteins. In this study, Anoikis-resistant is used as an operational term for long-term suspension-selected derivatives showing reduced detachment-associated apoptotic readouts relative to matched parental cells. This designation does not exclude contributions from altered proliferation, dormancy-like adaptation, or aggregate-mediated survival.

### Western blot

Cells were lysed using RIPA buffer containing 50 mM Tris-HCl (pH 8.0), 150 mM NaCl, 1% Triton X-100, 0.5% sodium deoxycholate, and 0.1% SDS, supplemented with protease and phosphatase inhibitor cocktails (Sigma-Aldrich). Lysates were incubated on ice for 10 min and centrifuged at 12,000 rpm for 15 min at 4 °C to remove cellular debris. Protein concentrations were quantified using the Pierce™ BCA Protein Assay Kit (Thermo Scientific).

Equal amounts of protein were mixed with 4 × Laemmli loading buffer (Bio-Rad), denatured at 95 °C for 5 min, and separated by SDS-PAGE using 4–20% Mini-PROTEAN® TGX™ precast gels (Bio-Rad) at 150 V for 40 min. Proteins were subsequently transferred onto PVDF membranes using the Trans-Blot Turbo transfer system (Bio-Rad) according to the manufacturer’s instructions.

After the transfer, the membranes were blocked in 5% non-fat milk in TBS-T for 1.5 h at room temperature and then incubated overnight at 4 °C with primary antibodies diluted in blocking buffer. The following primary antibodies were used: anti-cleaved-PARP rabbit polyclonal antibody (1:1000, Cell Signaling Technology) and anti-GAPDH mouse monoclonal antibody (1:1000, Merck Millipore). After washing with TBS-T, the membranes were treated with HRP-conjugated goat anti-rabbit or goat anti-mouse secondary antibodies (Sigma-Aldrich) for 1 h at room temperature. Protein bands were detected using SuperSignal™ West Femto Maximum Sensitivity Substrate (Thermo Scientific) and visualized with an Odyssey Fc Imaging System (LI-COR Biosciences).

### Flow cytometric analysis of apoptosis

Suspension cells were collected and centrifuged at 1200 rpm for 3 min. After removal of the supernatant, cells were dissociated using TrypLE™ Select Enzyme (Gibco) for 3–5 min at room temperature. Complete culture medium was then added to terminate digestion, followed by centrifugation at 1200 rpm for 3 min. Cells were washed twice with PBS and resuspended in 1 × binding buffer at a concentration of 1 × 10^6^ cells/mL.

For apoptosis analysis, 100 μL of cell suspension (1 × 10^5^ cells) was transferred into a 5-mL tube and stained using the FITC Annexin V Apoptosis Detection Kit I (BD Pharmingen™). Briefly, 5 μL Annexin V-FITC and 5 μL propidium iodide (PI) were added to each sample, followed by incubation in the dark for 15 min at room temperature. Subsequently, 400 μL of 1 × binding buffer was added to each tube prior to analysis. Samples were analyzed using a BD Accuri C6 flow cytometer (BD Biosciences). Data processing and statistical analysis were performed using FlowJo v10.8.1.

### RNA extraction

Total RNA was extracted from parental and anoikis-resistant (AR) cell pellets (three biological replicates per condition) using the RNeasy Plus Mini Kit (Qiagen) according to the manufacturer’s protocol. Cells were lysed in RLT Plus buffer. After gDNA elimination and ethanol adjustment, RNA was eluted in 30–50 µL of RNase‑free water. RNA concentration and purity were assessed by Nanodrop spectrophotometry (A260/A280 > 2.0 for all samples; A260/A230 ranged from 0.74 to 2.20). All samples were stored at –80 °C and subsequently delivered to The Center for Translational Genomic (Lund University) for downstream analysis.

### RNA sequencing and data processing

Raw paired‑end reads (150 bp) were trimmed with Trim Galore (v0.6.7; parameters: --quality 20 --phred33 --length 36), followed by quality assessment with FastQC (v0.11.9) and MultiQC (v1.14). Cleaned reads were aligned to the human reference genome (GRCh38) using STAR (v2.7.10a), and transcript quantification was performed with Salmon (v1.10.0) to obtain TPM and raw count matrices. The pipeline was built on nf-core/rnaseq (v3.x), and software versions are provided in the MultiQC report.

### Gene set variation analysis (GSVA)

The following analysis was conducted using R (v.4.5.3). Raw gene counts were normalized using the trimmed mean of M‑values (TMM) method implemented in edgeR (v3.38.4) to correct for sequencing depth and RNA composition. Normalized log₂ counts per million (log₂CPM) with a prior count of 1 were calculated and used as input for Gene Set Variation Analysis (GSVA). Canonical pathway (CP) gene sets were extracted from the MSigDB collection c2.all.v2026.1.Hs.symbols.gmt by retaining identifiers starting with KEGG, REACTOME, PID, BIOCARTA, or CP. Pathways containing fewer than 5 or more than 500 genes were excluded to avoid unstable estimates and overly broad terms. GSVA (v1.44.5) was performed using the gsvaParam method with a Gaussian kernel.

### Differential pathway activity for individual comparisons

For each comparison (Capan-1 AR vs Capan-1 parental and PSN-1 AR vs PSN-1 parental), differential pathway activity was assessed using limma (v3.52.4). A linear model was fitted (~ 0 + Group), the contrast of interest was extracted, and empirical Bayes moderation (eBayes) was applied. Adjusted p‑values were calculated using the Benjamini‑Hochberg method. A |log₂ fold change | ≥ 0.5 and adjusted p‑value < 0.05 was considered significant. For each comparison, significantly upregulated and downregulated pathways were ranked by absolute log₂FC, and the top 15 pathways in each direction were visualized as bar plots, where bar length represents log₂FC and color intensity represents –log₁₀(adjusted p‑value).

### Common pathway identification and ranking

Pathways that were significantly upregulated or downregulated in both AR-versus-parental comparisons and showed the same direction of change were defined as common pathways. This definition was used to identify candidate transcriptional programs showing cross-model concordance between the two biologically distinct PDAC cell lines.

For exploratory prioritization of these already concordant pathways, a composite ranking score was calculated as:

$$\begin{array}{c}{\rm{Score}}=|\log \,2{\rm{FC}}\_{\rm{Capan}}-1|\times (-\,\log \,10\,{\rm{adjusted}}\,{\rm{P}}\_{\rm{Capan}}-1)\\ +|\log \,2{\rm{FC}}\_{\rm{PSN}}-1|\times (-\,\log \,10\,{\rm{adjusted}}\,{\rm{P}}\_{\rm{PSN}}-1).\end{array}$$

This score was used solely as a descriptive ranking metric and was not intended as a pooled effect size, formal meta-analysis, or statistical test of equivalence between cell lines. Cell-line-specific log2 fold changes and adjusted P values were retained and reported separately.

### Differential expression analysis

Differential expression analysis was conducted using DESeq2 (v1.38.3) for the comparisons of gene expressions in Capan-1 AR vs Capan-1 parental and PSN-1 AR vs PSN-1 parental cells. Volcano plots were generated for each comparison using ggplot2. Cutoff lines were drawn at |log₂FC| = 1 and adjusted p‑value = 0.05. To assess overlap of significant genes between Capan-1 AR vs Capan-1 and PSN-1 AR vs PSN-1, pairwise Venn diagrams were drawn using VennDiagram.

### Gene Ontology (GO) biological process enrichment analysis

GO BO analysis was performed using the hypergeometric test implemented in R. Differentially expressed genes (DEGs) were defined as those with |log₂ fold change | ≥ 1 and adjusted p‑value (Benjamini-Hochberg) < 0.05.

For each comparison (Capan-1 AR vs Capan-1 and PSN-1 AR vs PSN-1), upregulated and downregulated genes were analyzed separately against the GO Biological Process (BP) gene sets (c5.all.v2026.1.Hs.symbols.gmt, Molecular Signatures Database). Only terms with at least 3 overlapping genes and an adjusted p‑value < 0.05 were considered significantly enriched.

Pathways detected in Capan-1 AR vs Capan-1 and PSN-1 AR vs PSN-1 were defined as common when significantly enriched in the same direction (both upregulated or both downregulated) in both comparisons. Dot plots were generated using ggplot2 to visualize gene ratio, gene count, and adjusted p‑value. Package “GSEABase”, “dplyr”, and “ggplot2” was used.

### Survival analysis and Cox regression

Survival analysis was performed using an independent clinical cohort, described previously [19]. Gene expression data were obtained from the same RNA‑seq dataset after ComBat batch correction and log₂(CPM + 1) transformation. Clinical data included overall survival (OS) in days and vital status. OS was truncated at 5 years. For each gene of interest, the optimal expression cutoff to divide patients into high‑ and low‑expression groups was determined using the maxstat method with log‑rank test, restricting cutoffs to the 30‑70% percentile range to ensure balanced group sizes.

Univariate Cox proportional hazards regression was then applied to estimate the hazard ratio (HR) and its 95% confidence interval (CI) for 5‑year OS, comparing high vs. low expression groups. Kaplan‑Meier curves were plotted for genes with HR > 1 and p < 0.05, with log‑rank p‑values reported. All statistical tests were two‑sided, and p < 0.05 was considered significant. Analyses were performed using R packages “survival”, “survminer”, and “maxstat”.

## Results

### Anoikis-resistant pancreatic cancer cell lines

**Fig. 1:**
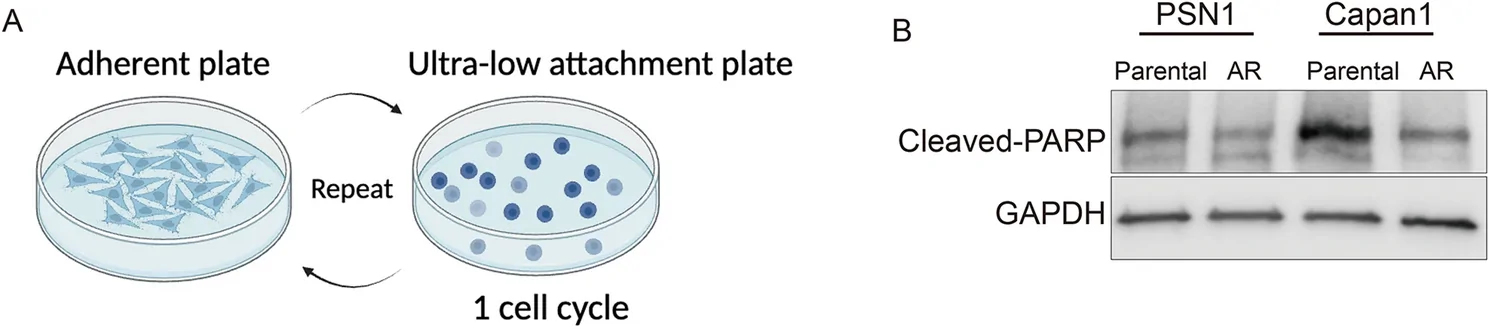
Development and validation of anoikis-resistant pancreatic cancer cell lines (**A**) Representative morphology of parental and AR pancreatic cancer cells under detached culture conditions. **B** Western blot analysis of cleaved-PARP expression in parental and AR pancreatic cancer cell lines under detached conditions. GAPDH was used as a loading control. Original unprocessed raw image files of the western blots are provided as supporting information

To investigate the molecular alterations associated with anoikis resistance in pancreatic cancer, we established anoikis-resistant (AR) cell lines through repeated cycles of suspension and re-attachment culturing conditions (Fig. 1A). 

**Fig. 2:**
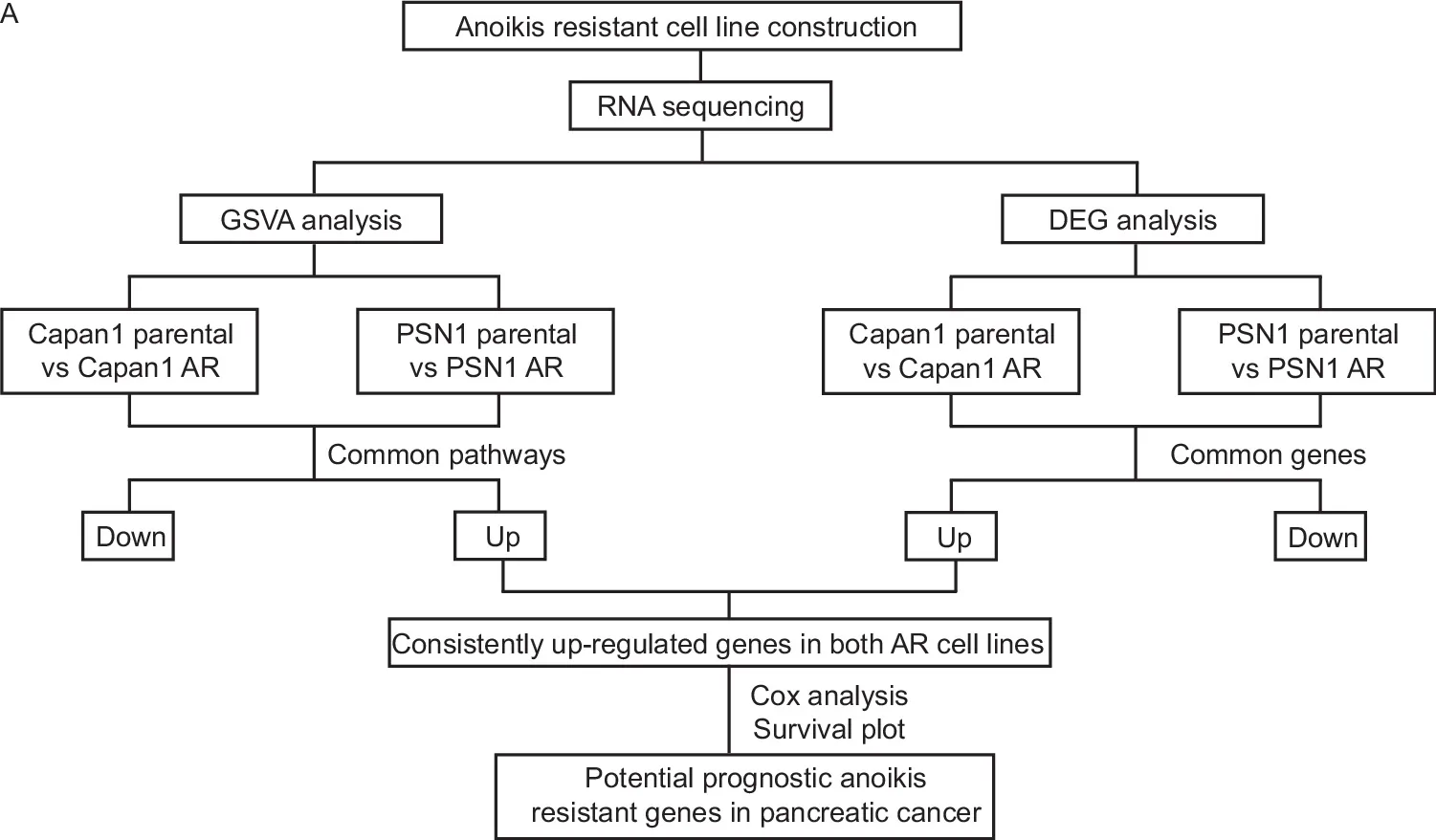
Schematic workflow (**A**) Schematic workflow illustrating the establishment of anoikis-resistant (AR) pancreatic cancer cell lines and the subsequent bioinformatic analysis pipeline

Western-blot experiments showed that anoikis-resistant pancreatic cell lines expressed lower levels of cleaved-PARP in detached culturing environment (Fig. 1B and Supplementary Fig. 1). Flow cytometry analysis for PSN-1 cell group was conducted to provide orthogonal support (Supplementary Fig. 2). The workflow is demonstrated in Fig. 2.

### RNA-seq data quality assessment

**Fig. 3:**
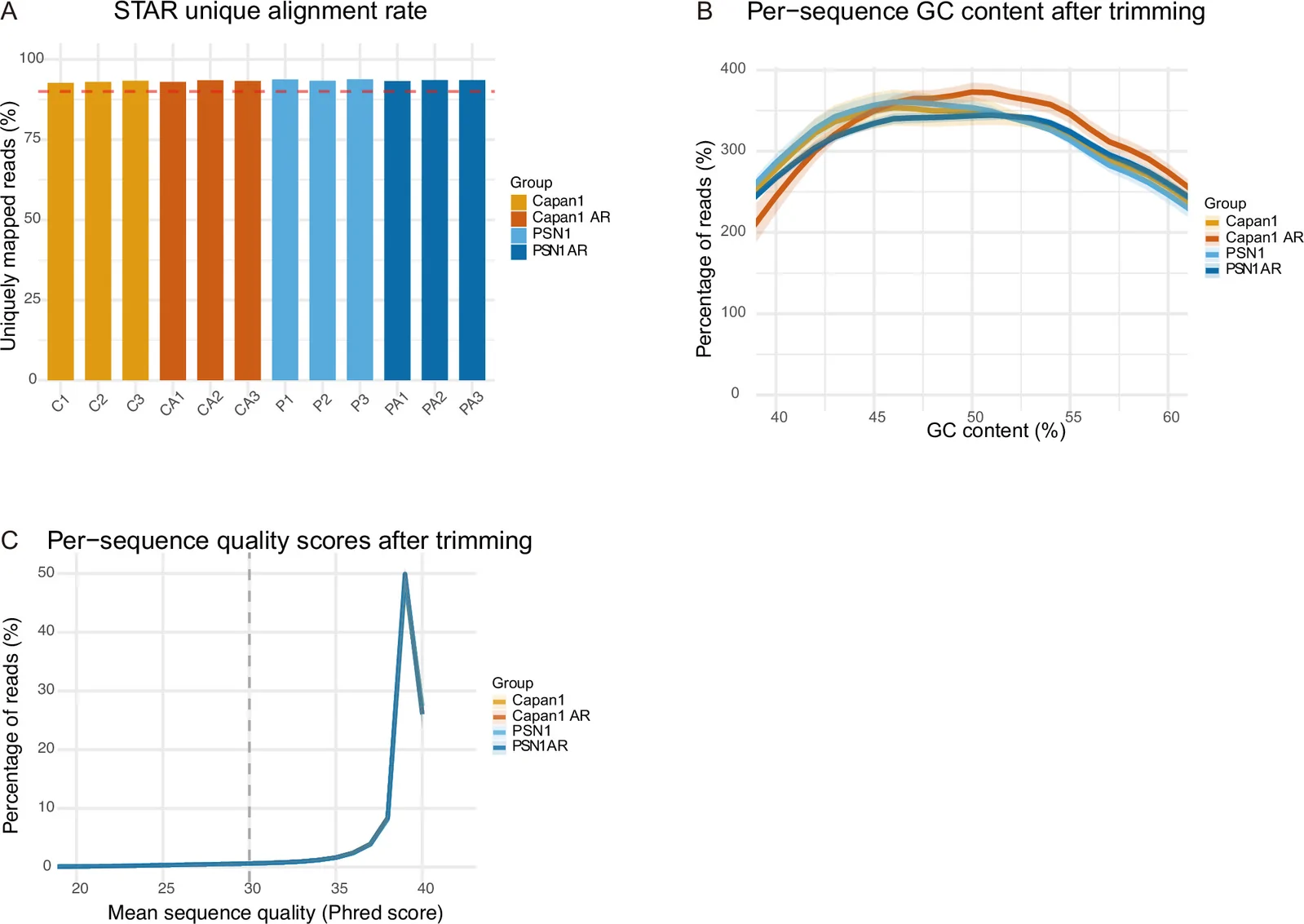
Quality assessment of RNA sequencing data from parental and anoikis-resistant pancreatic cancer cells. **A** STAR unique alignment rates of RNA-seq reads in Capan-1, Capan-1 AR, PSN-1, and PSN-1 AR samples. **B** Distribution of GC content after trimming across all sequencing samples. **C** Distribution of mean sequence quality (Phred scores) after trimming among all groups

Raw read counts per sample ranged from 49.1 M to 65.3 M, with overall alignment rates to GRCh38 of 98.4–98.8% and unique mapping rates of 92.7–93.8% (Fig. 3A). No rRNA contamination was detected. Duplication rates ranged from 18.1–26.5%, and the proportion of proper pairs was 60.9–70.5%. GC content of trimmed reads showed an approximately normal distribution (main peak 50–52%), with highly overlapping curves across groups, indicating uniform libraries and absence of AT/GC bias (Fig. 3B). Average quality scores exhibited a unimodal distribution peaking at Q37-Q38; the percentage of reads with Q > 30 ranged from 95.99–96.97% (mean > 96.5%) (Fig. 3C). These metrics confirmed our sequencing data is suitable for downstream differential expression analysis.

### Gene set variation analysis and common regulated pathways

To obtain a more complete and unbiased functional landscape of the two cell lines, we first applied Gene Set Variation Analysis (GSVA) to guide subsequent differential analyses. Significantly altered pathways were identified with adjusted p < 0.05.

### Capan-1 AR vs Capan-1 parental

**Fig. 4:**
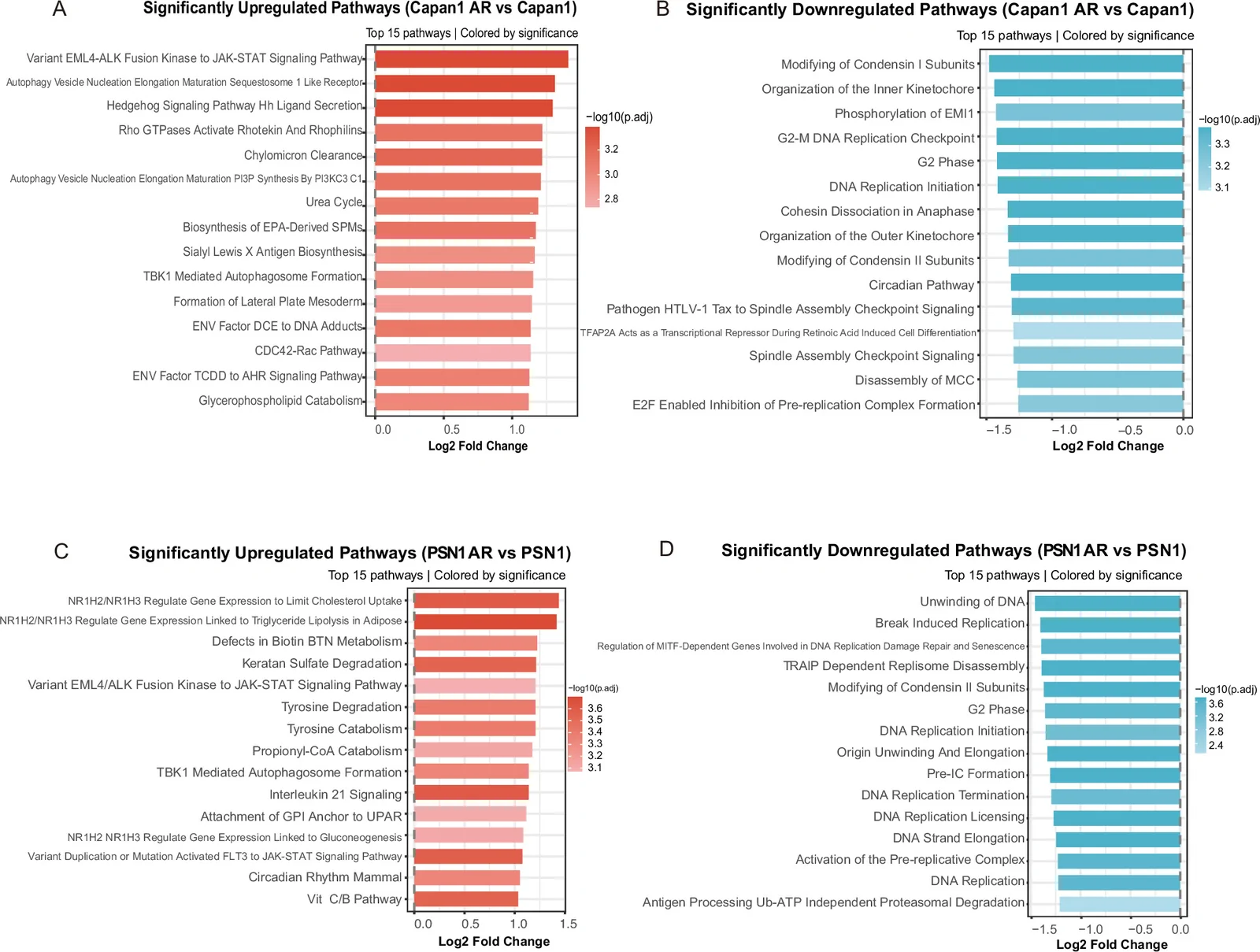
GSVA pathway analysis of anoikis-resistant pancreatic cancer cells. **A**,** B** Top 15 significantly upregulated and downregulated pathways in Capan-1 AR cells compared with parental Capan-1 cells. **C**, **D** Top 15 significantly upregulated and downregulated pathways in PSN-1 AR cells compared with parental PSN-1 cells

Compared with Capan-1 parental cells, Capan-1 AR cells exhibited significant upregulation of pathways associated with metabolic reprogramming and cellular stress responses. As shown in Fig. 4A, autophagy‑related pathways were markedly transcriptomically enriched, including autophagy vesicle nucleation and elongation and PI3KC3-mediated autophagy. The Hedgehog signaling pathway and Rho GTPase signaling pathway were also significantly enriched among the top 15 upregulated pathways, indicating altered survival and cytoskeletal regulatory programs. Furthermore, the 1,2-dichloroethane (DCE)-to-DNA-adducts pathway was significantly enriched within the top 15. These findings suggest that Capan-1 AR cells may experience elevated oxidative stress or xenobiotic exposure, accompanied by enrichment of metabolic reprogramming, autophagy, and stress responses involving DNA adduct formation.

In contrast, downregulated pathways in Capan-1 AR cells presented in Fig. 4B were predominantly associated with cell cycle progression and DNA damage repair. Additional downregulated pathways were related to kinetochore organization and condensin subunit modification. These results indicate that Capan-1 AR cells may exhibit G2/M cell cycle arrest, impaired mitotic progression and compromised DNA repair capacity.

### PSN-1 AR vs PSN-1 parental

Compared with PSN-1 parental cells, PSN-1 AR cells exhibited significant upregulation of pathways associated with metabolic reprogramming and stress‑related signaling. As shown in Fig. 4C, the top 15 upregulated pathways were related to nuclear receptor regulation of lipid and glucose metabolism including keratan sulfate degradation, tyrosine degradation, and propionyl‑CoA catabolism. Autophagy‑related vesicle formation, JAK‑STAT signaling and the vitamin C pathway were also among the top upregulated pathways.

Equivalently to Capan-1 AR cells, top downregulated pathways in PSN-1 AR cells were predominantly associated with DNA replication, cell cycle progression, and replisome assembly as presented in Fig. 4D. These findings further indicate that AR pancreatic cancer cells exhibit impaired DNA replication initiation and elongation, compromised replisome stability, and G2 phase arrest.

### Common GSVA pathways

**Fig. 5:**
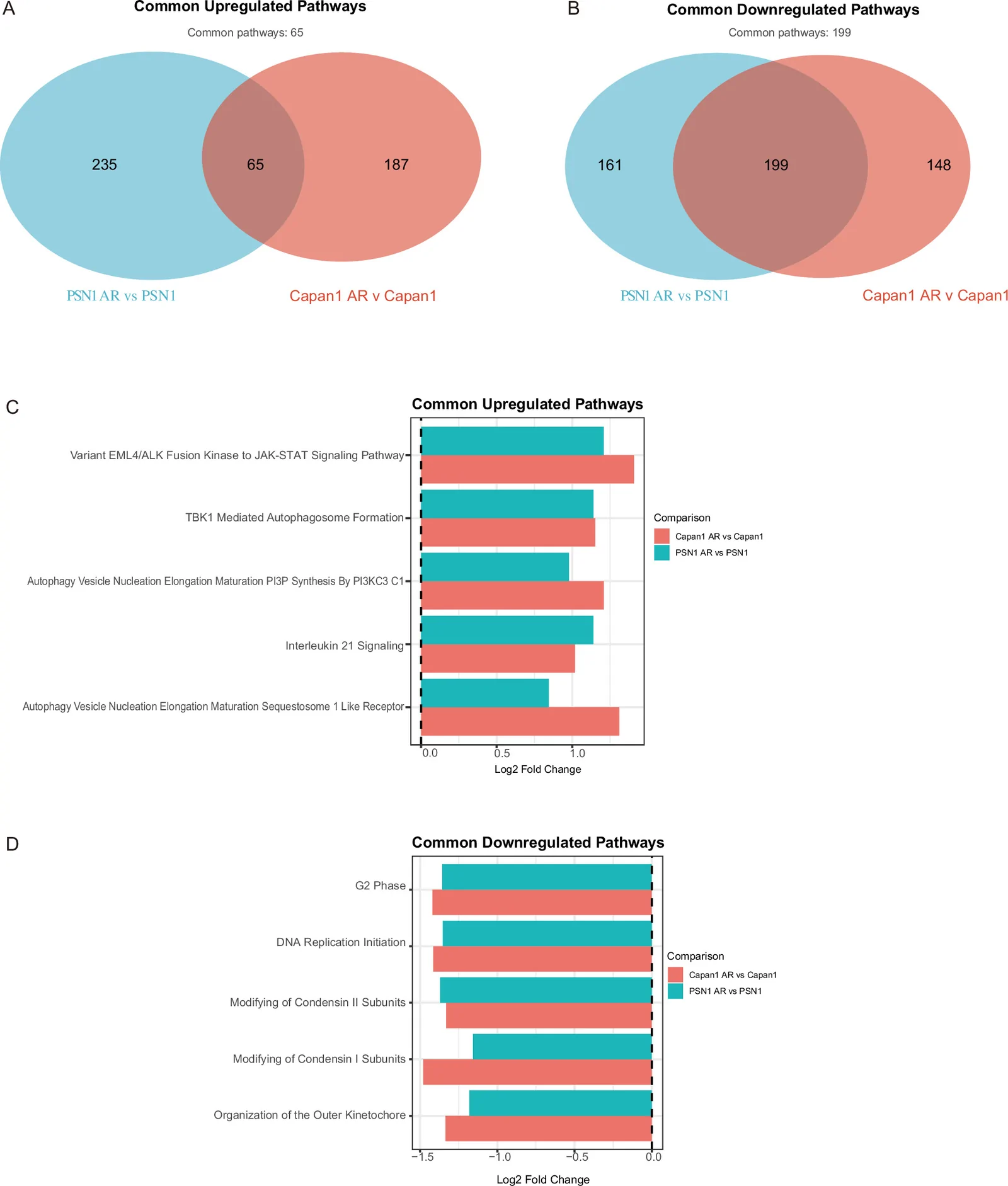
Identification of common altered pathways in anoikis-resistant pancreatic cancer cells. **A**,** B** Venn diagram showing commonly upregulated/downregulated pathways between Capan-1 AR vs. Capan-1 and PSN-1 AR vs. PSN-1 comparisons. Central values indicate pathways significantly altered in the same direction in both comparisons; outer values indicate pathways specific to each cell-line comparison. **C**,** D** Top 5 significantly and commonly upregulated/ downregulated pathways identified in both AR cell lines

To identify the core biological alterations shared by AR pancreatic cancer cells, we focused on pathways altered across both cell line groups. Overall, 252 (65 + 187) pathways were found significantly upregulated in Capan-1 AR cells and 300 (235 + 65) in PSN-1 AR cells. 347 (199 + 148) down-regulated pathways were observed in Capan-1 AR cells and 360 (161 + 199) pathways in PSN-1 AR cells. In both cell lines, 65 commonly up-regulated and 199 commonly down-regulated pathways were identified (Fig. 5A, B).

To prioritize commonly dysregulated pathways, we defined a ranking score for each pathway as the sum of the product of absolute log₂ fold change and the negative logarithm of the adjusted p‑value from the two comparisons: Higher GSVA scores indicating stronger and more consistent pathway alterations across both contrasts.

Top‑5 upregulated pathways were enriched in autophagy (e.g., autophagosome formation, TBK1‑mediated autophagy) and immune signaling (EML4‑ALK/JAK‑STAT, IL‑21 signaling). Top‑5 downregulated pathways were enriched in cell cycle progression (G2 phase, DNA replication initiation) and chromosome segregation (condensin I/II subunits, outer kinetochore organization) (Fig. 5C, D).

Taken together, Capan-1 AR and PSN-1 AR cells showed transcriptomic enrichment of autophagic activity and JAK‑STAT/IL‑21 signaling, along with reduced cell‑cycle and mitotic apparatus pathways, suggesting a transcriptional state that contribute to anoikis resistance.

### Differentially expressed genes analysis

**Fig. 6:**
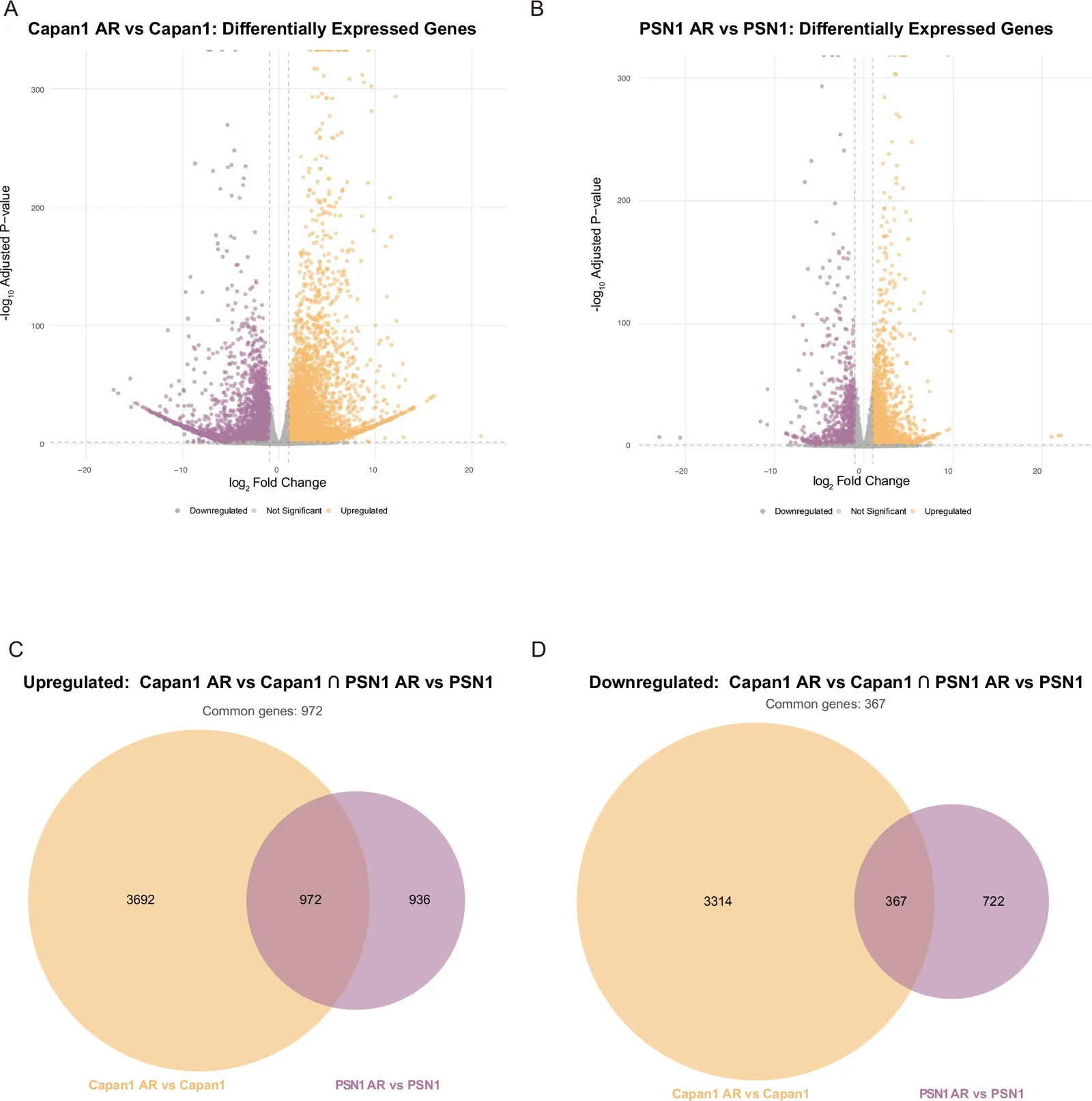
Differential gene expression analysis in anoikis-resistant pancreatic cancer cells. **A**,** B** Volcano plots of DEGs in Capan-1 AR and PSN-1 AR cells relative to their respective parental cells. **C**,** D** Venn diagrams showing the intersection of upregulated (**C**) and downregulated (**D**) DEGs identified in the Capan-1 AR vs. parental and PSN-1 AR vs. parental cells comparisons

Next performed differentially expression analysis between AR cells and parental cells to further identify individual genes associated with anoikis resistance. As shown in the volcano plot, 4664 genes were significantly upregulated and 681 genes were significantly downregulated in Capan-1 AR cells compared to Capan-1 parental cell. In PSN-1 AR cells, 1908 genes were upregulated and 1089 genes were downregulated compared to PSN-1 parental cells (|log2FC | ≥ 1 and adjusted p < 0.05) (Fig. 6A, B). Cross-comparison analysis identified 972 commonly upregulated genes and 367 commonly downregulated genes shared by both AR models (Fig. 6C, D).

**Fig. 7:**
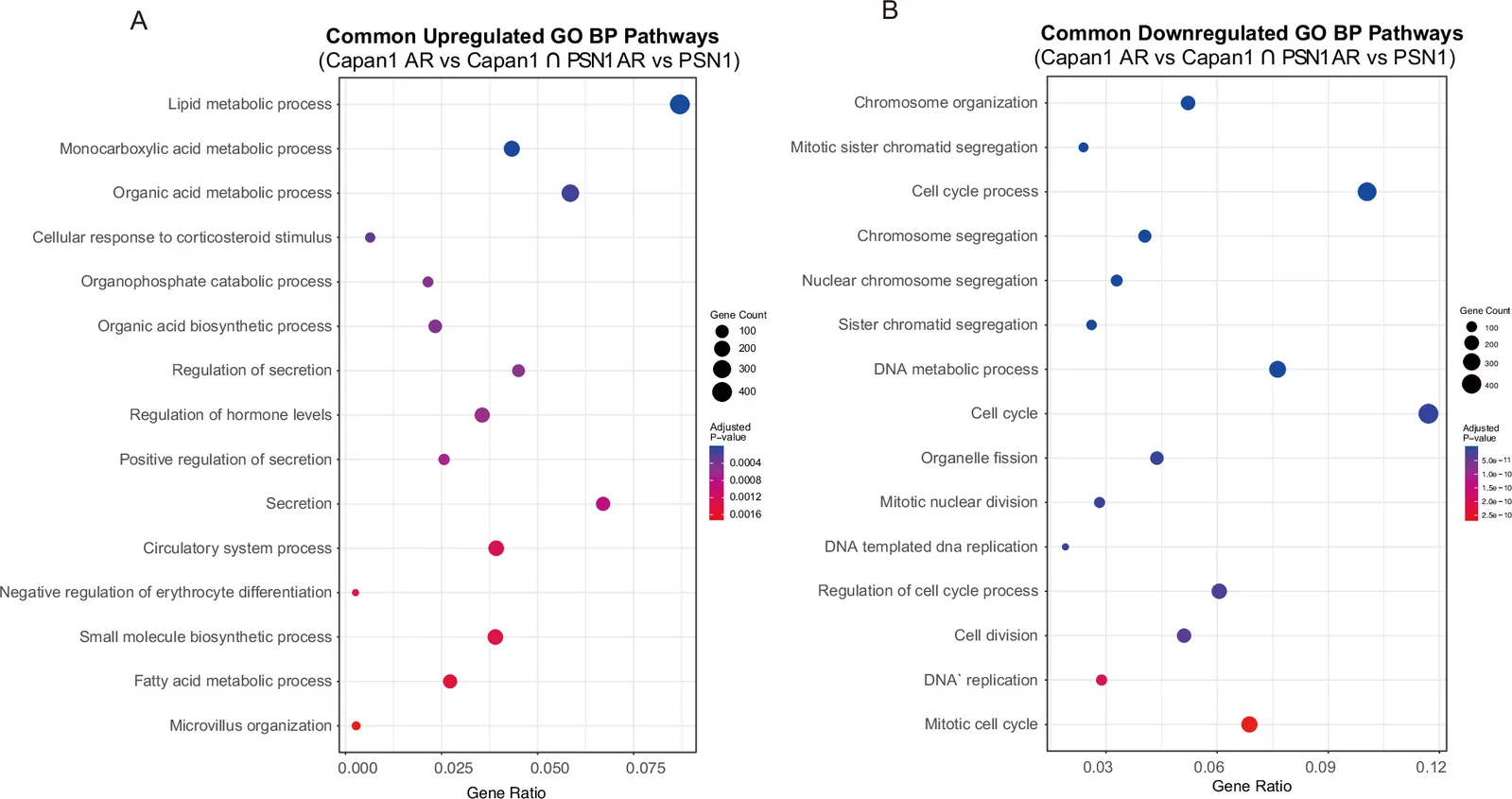
GO biological process enrichment analysis of common differentially expressed genes in anoikis-resistant pancreatic cancer cells. **A** GO biological process enrichment analysis of commonly upregulated genes shared between the two AR cell lines comparison groups. **B** GO biological process enrichment analysis of commonly downregulated genes shared between the two AR cell lines comparison groups

Gene Ontology (GO) biological process (BP) enrichment analysis demonstrated that commonly upregulated genes were primarily associated with metabolic remodeling and secretory regulation, including lipid metabolic processes, organic acid metabolism, and secretion-related pathways (Fig. 7A). In contrast, commonly downregulated genes were strongly enriched in pathways related to chromosome organization, mitotic progression, and DNA replication (Fig. 7B). These findings further support the notion that AR pancreatic cancer cells undergo coordinated metabolic adaptation while suppressing proliferative programs.

### Integration of pathway‑level and single‑gene level changes

To identify transcriptional programs showing concordant changes across two distinct PDAC models, we first identified pathways that were significantly altered in the same direction in both cell-line-specific AR-versus-parental comparisons. These common pathways were interpreted as candidate cross-model conserved programs, while recognizing that the magnitude and biological basis of the changes may differ between PSN-1 and Capan-1.

We then used an exploratory composite ranking score to prioritize the already concordant pathways for visualization. This ranking was not used to estimate a pooled effect or to infer statistical equivalence between the two cell lines (Table 1).

**Table 1:** Candidate genes identified through differential expression analyses in AR PDAC cells and integrated GSVA

Gene	logFC Capan-1 group	Padj Capan-1 group	logFC PSN-1 group	Padj PSN-1 group	GSVA Pathway
JAK3	2.9408	3.77E-30	3.6683	3.62E-44	Variant EML4/ALK Fusion Kinase to JAK-STAT Signaling Pathway/Interlukin-21 Signaling
STAT5A	4.5137	1.21E-186	2.0031	2.83E-37	Variant EML4/ALK Fusion Kinase to JAK-STAT Signaling Pathway/Interlukin-21 Signaling
STAT5B	2.2955	6.13E-68	0.8686	4.11E-16	Variant EML4/ALK Fusion Kinase to JAK-STAT Signaling Pathway/Interlukin-21 Signaling
STAT3	1.6602	6.92E-18	0.7065	2.48E-10	Variant EML4/ALK Fusion Kinase to JAK-STAT Signaling Pathway/Interlukin-21 Signaling
WIPI1	3.6500	3.62E-81	0.8362	1.45E-11	Autophagy Vesicle Nucleation Elongation Maturation PI3P Synthesis by PI3KC3-C1
ZFYVE1	2.2091	3.57E-30	1.1454	3.24E-20	Autophagy Vesicle Nucleation Elongation Maturation PI3P Synthesis by PI3KC3-C1
WDR45	1.3937	1.53E-30	0.7745	7.98E-17	Autophagy Vesicle Nucleation Elongation Maturation PI3P Synthesis by PI3KC3-C1
ATG14	1.4246	3.71E-29	0.6163	1.12E-09	Autophagy Vesicle Nucleation Elongation Maturation PI3P Synthesis by PI3KC3-C1
NBR1	2.5928	4.83E-31	1.1279	2.58E-35	TBK1 Mediated Autophagosome Formation/Autophagy Vesicle Nucleation Elongation Maturation Sequestosome 1 Like Receptor
CALCOCO2	1.8314	7.82E-32	0.5540	2.73E-10	TBK1 Mediated Autophagosome Formation/Autophagy Vesicle Nucleation Elongation Maturation Sequestosome 1 Like Receptor
UBC	0.6101	0.012691961	0.6381	1.00E-06	Autophagy Vesicle Nucleation Elongation Maturation Sequestosome 1 Like Receptor

Thes obtained set of genes clustered into two functional categories: JAK‑STAT signaling (JAK3, STAT3, STAT5A, STAT5B) and autophagy‑related processes (WIPI1, ZFYVE1, WDR45, ATG14, NBR1, CALCOCO2, UBC). The intersection thus highlights the two most robustly transcriptomically enriched functional modules associated with anoikis resistance and provides a shortlist of candidate drivers associated with these enriched transcriptional programs.

### Prognostic analysis of core anoikis-resistant related genes

**Fig. 8:**
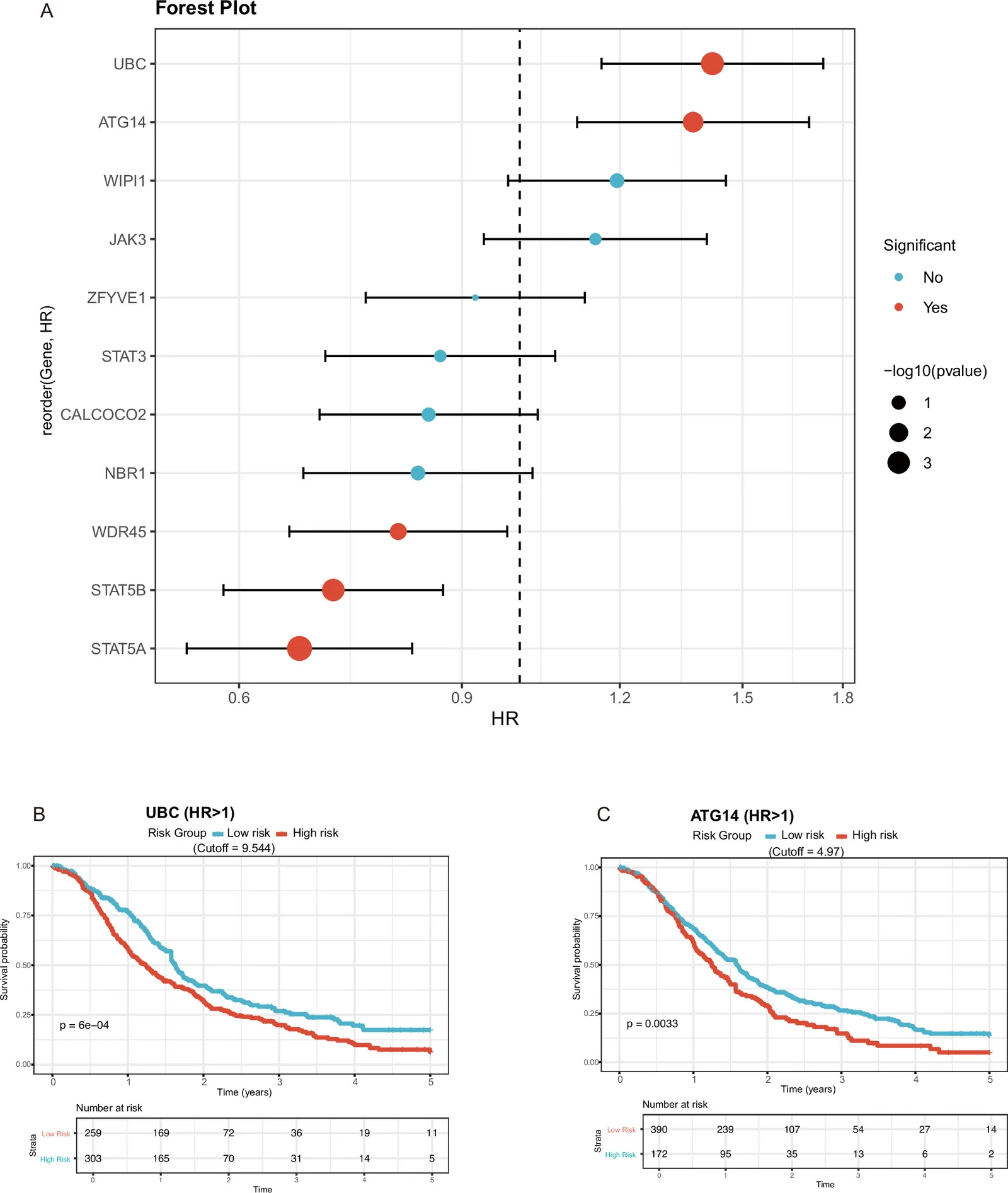
Prognostic analysis of autophagy-related genes in pancreatic cancer. **A** Forest plot showing hazard ratios (HRs) of candidate autophagy-related genes associated with overall survival in pancreatic cancer patients. **B** Kaplan–Meier survival analysis stratified by ATG14 expression levels. **C** Kaplan–Meier survival analysis stratified by UBC expression levels

We assessed the association between expression levels of the 11 core upregulated genes and 5‑year overall survival (OS) in a clinical cohort. Univariate Cox regression identified five genes with statistically significant prognostic value (p < 0.05) (Fig. 8A).

Among these, UBC (Ubiquitin C) (HR = 1.42, 95% CI: 1.16–1.74, p = 6.60 × 10^−4^) and ATG14 (Autophagy Related 14) (HR = 1.37, 95% CI: 1.11–1.69, p = 0.0034) demonstrated HR > 1, indicating that higher expression is linked to poorer prognosis (Fig. 8B, C). In contrast, STAT5A, STAT5B, and WDR45 showed HR < 1, suggesting that higher expression of the genes is associated with favorable prognosis. These results propose that UBC and ATG14 may serve as adverse prognostic indicators in the context of anoikis resistance, while STAT5A, STAT5B, and WDR45 are potential protective markers.

## Discussion

In this study, we established anoikis‑resistant pancreatic models from PSN-1 and Capan-1 pancreatic cancer cell lines representing primary and metastatic disease respectively. Further, we performed comparative transcriptomic analysis and identified conserved adaptive programs associated with anoikis resistance and patient prognosis. This approach increases the robustness of our findings and reduces the likelihood that observed adaptations are specific to a single cellular context or restricted to a specific stage of disease progression. We performed GSVA to obtain an overview at the pathway level of the functional differences between anoikis-resistant and parental cells, before examining individual differently expressed genes (DEGs) and thereby reducing reliance on individual genes with large expression changes.

Because pathway enrichment and DEG analyses provide complementary information regarding transcriptomic regulation, we intersected genes from commonly enriched GSVA pathways with genes consistently upregulated in both anoikis-resistant models.

This approach generated a functionally anchored gene set, likely representing candidate mediators of the shared anoikis‑resistant phenotype. 

**Fig. 9:**
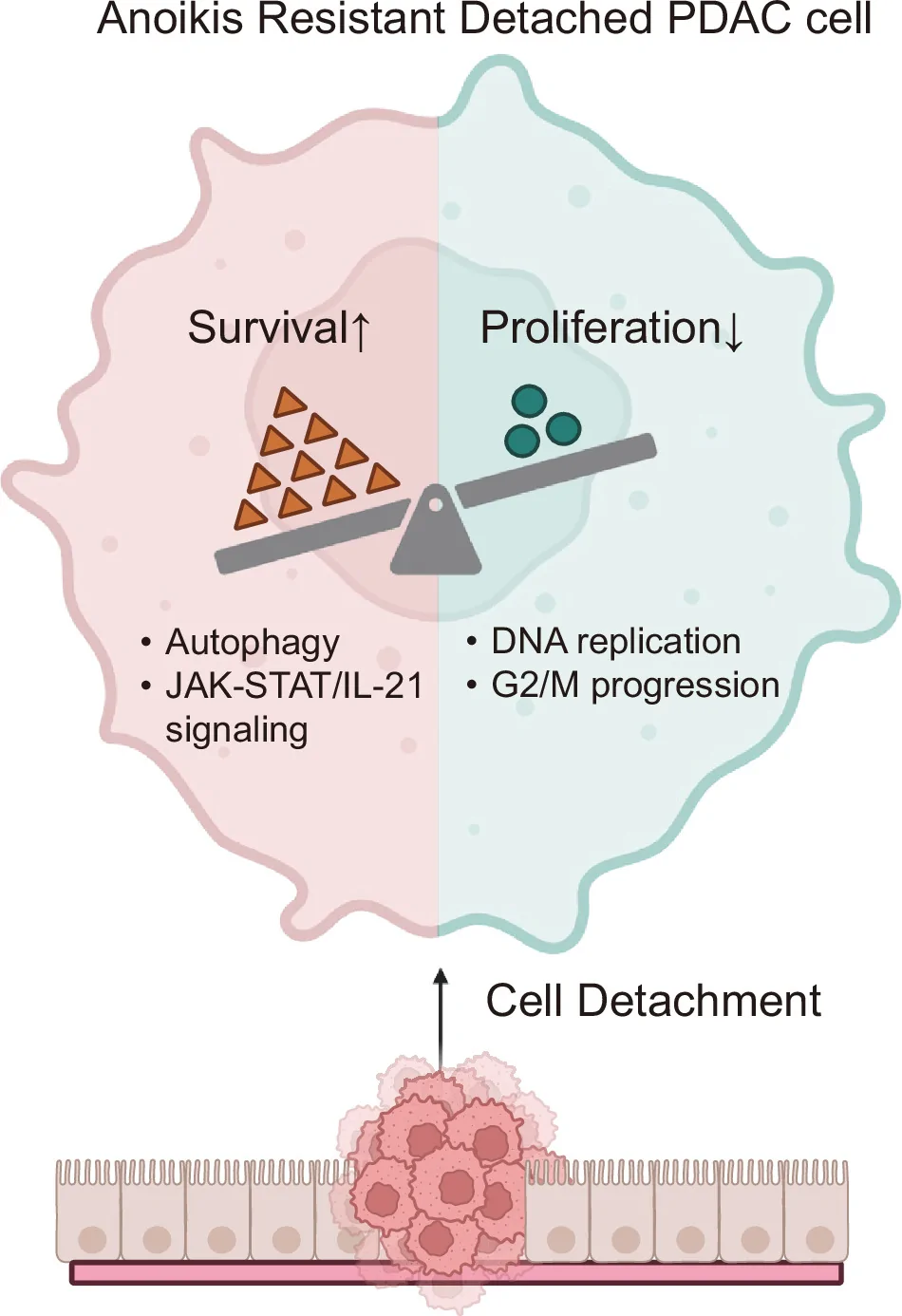
Proposed survival-proliferation trade-off model in anoikis-resistant pancreatic cancer cells

Although the identification of concordantly altered pathways across PSN-1 and Capan-1 provides a conservative strategy for prioritizing candidate programs across distinct PDAC backgrounds, it does not imply that the two models share identical baseline biology or equivalent AR-associated effect sizes. Cell-line-specific adaptations may be particularly relevant given the primary versus metastatic origin of the two cell lines. Despite the different origin, both anoikis-resistant cell lines exhibited consistent downregulation of pathways involved in DNA replication, chromosome segregation, and G2/M progression. This transcriptional profile is consistent with a stress-adaptive, dormancy-like state that may facilitate tumor cell survival under anchor independent conditions, similar to adaptations observed in disseminated metastatic cells during matrix deprivation or nutrient stress. Pancreatic anoikis- resistant cells may additionally activated pro-survival mechanisms, notably JAK–STAT signaling (STAT5A/B, JAK3) and autophagy-related genes (WIPI1, ATG14, NBR1). Together, these changes may reflect a trade-off where cells temporarily suspend proliferation to sustain survival and stress adaptation (Fig. 9).

Autophagy-associated pathways were consistently enriched in both resistant models. Detached tumor cells experience substantial metabolic and oxidative stress due to loss of ECM-dependent signaling and impaired nutrient access [13]. Under these conditions, enhanced autophagy may provide an essential survival advantage by maintaining ATP production, removing damaged organelles, and limiting stress-induced apoptosis [20, 21]. Previous studies have shown that pancreatic cancer cells are highly dependent on autophagy for metabolic fitness [22–24]. Our findings further suggest that the autophagy-associated transcriptional programs are enriched in anoikis-resistant cells under detachment stress.

Among the commonly upregulated genes, UBC and ATG14 were particularly notable for the association with poor patient survival. UBC encodes polyubiquitin, and ubiquitin functions as a central regulator of protein turnover through ubiquitin–proteasome system [25, 26]. Although ubiquitin signaling has been widely implicated in pancreatic cancer progression [27–29], direct investigation of the polyubiquitin gene UBC in PDAC remains limited. Our results showed that UBC was consistently upregulated in AR pancreatic cancer cells, suggesting a potential role in maintaining ubiquitin homeostasis and supporting proteostatic adaptation during detachment survival [30].

ATG14 is a critical component of the PI3KC3‑C1, which initiates autophagosome nucleation [31]. Recent studies have shown that ATG14 is regulated in pancreatic cancer and contributes to autophagy-associated tumor survival, including the USP1–ATG14 axis that promotes autophagy and tumor progression [32]. In addition, the PVT1-miR-619-5p-ATG14 regulatory axis has been reported to enhance autophagy and contribute to gemcitabine resistance in pancreatic cancer [33]. In line with these findings, ATG14 upregulation in anoikis-resistant pancreatic cancer cells may reflect enhanced autophagy initiation capacity as part of an adaptive response to detachment-induced stress.

Importantly, this study relies on transcriptomic profiling and does not establish a causal role for the identified candidate genes or pathways in detachment survival. Several limitations should be considered. First, the anoikis-resistant phenotype was defined operationally; our in vitro suspension model cannot fully distinguish intrinsic anoikis resistance from alternative survival strategies, such as altered proliferation, aggregate-mediated protection, or general stress adaptation. Consequently, these in vitro findings do not serve as direct evidence of in vivo metastatic competence. Second, functional validations via genetic or pharmacological perturbations, alongside direct measurements of autophagic flux, are necessary to confirm the mechanistic roles of candidate programs like ATG14, UBC, and JAK-STAT signaling. Third, while utilizing two distinct PDAC cell lines (primary vs. metastatic) highlights conserved adaptive pathways, it does not imply identical baseline biology or effect sizes across different stages of tumor progression. Finally, the prognostic associations for ATG14 and UBC remain exploratory. Because these analyses were univariate and based on bulk-tumor RNA-seq from a single cohort without comprehensive clinical covariates, independent multivariable studies adjusting for factors such as metastatic status are required to validate their clinical utility.

In conclusion, our transcriptomic analyses identified candidate autophagy-, ubiquitin-, and JAK–STAT-associated programs that consistently correlated with long-term suspension survival in two PDAC cell models. The exploratory survival associations of ATG14 and UBC highlight these genes for further investigation, though functional, in vivo, and independent clinical validation studies remain essential to determine their biological and translational relevance.

## Supplementary information

Supplementary Fig legends

Supplementary_fig_1

Supplementary_fig_2

## Data Availability

The datasets generated and/or analysed during the current study are available in the Genome Sequence Archive (GSA) repository, https://ngdc.cncb.ac.cn/omix/preview/mOGndKNC.

## Abbreviations

AR: Anoikis-resistant
ATCC: American Type Culture Collection
BP: Biological Process
CI: Confidence Interval
CP: Canonical Pathway
DEG: Differentially Expressed Gene
ECM: Extracellular Matrix
GO: Gene Ontology
GSVA: Gene Set Variation Analysis
HR: Hazard Ratio
OS: Overall Survival
PDAC: Pancreatic Ductal Adenocarcinoma
PI: Propidium Iodide
RNA-seq: RNA Sequencing
TMM: Trimmed Mean of M-values
TPM: Transcripts Per Million
UPS: Ubiquitin–Proteasome System
