# Supplementary material for: Transcriptomic profiling reveals autophagy-associated programs and prognostic biomarkers in anoikis-resistant pancreatic cancer cells
Source: BMC Cancer. 2026 Oct 13;26:1241. doi: 10.1186/s12885-026-17107-7 (PMC13675964; doi:10.1186/s12885-026-17107-7)
Supplement: Supplementary file 1 [file 12885_2026_17107_MOESM1_ESM.pdf]

Supplementary Fig. 1 Original unprocessed raw image files of the western blots.

Supplementary Fig. 2 Characterization and morphological features of AR cells.
(A) Anoikis rates of PSN1 parental and PSN1 AR cells following 48h low-attachment culture, as determined by flow cytometry. (B) Bright-field images (4× objective) showing the detached morphology of PSN1 AR and Capan1 AR cells. Scale bars are not available in these images, and thus the images are intended for qualitative morphological comparison only.
