# Supplementary material for: Transcriptomic profiling reveals autophagy-associated programs and prognostic biomarkers in anoikis-resistant pancreatic cancer cells
Source: BMC Cancer. 2026 Oct 13;26:1241. doi: 10.1186/s12885-026-17107-7 (PMC13675964; doi:10.1186/s12885-026-17107-7)
Supplement: Supplementary file 2 [file 12885_2026_17107_MOESM2_ESM.pdf]

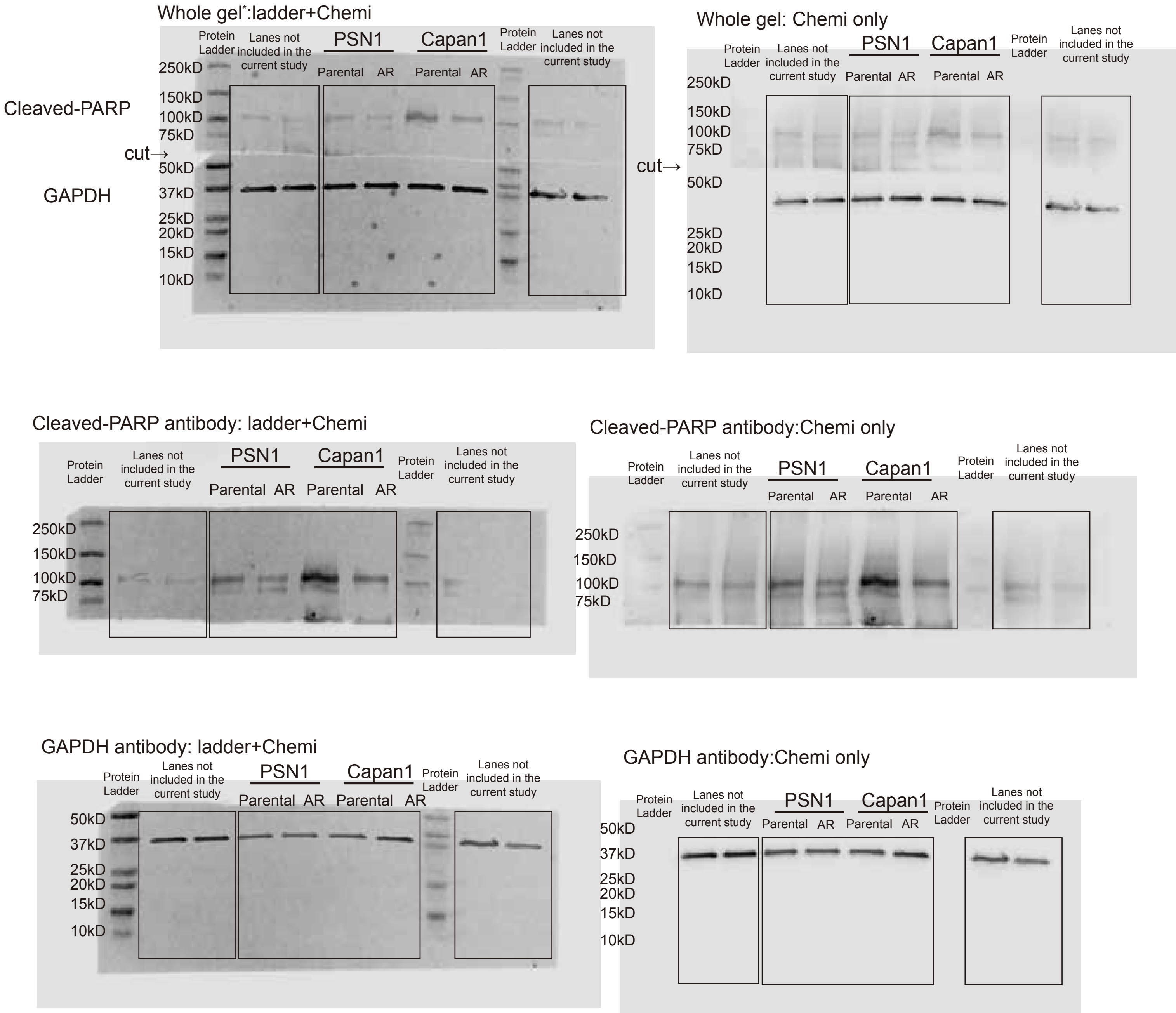

*Membranes were generated from the same gel and cut according to molecular weight prior to antibody incubation.
Images from different membrane sections were reassembled and developed in the scanner.
