# Supplementary material for: Transcriptomic profiling reveals autophagy-associated programs and prognostic biomarkers in anoikis-resistant pancreatic cancer cells
Source: BMC Cancer. 2026 Oct 13;26:1241. doi: 10.1186/s12885-026-17107-7 (PMC13675964; doi:10.1186/s12885-026-17107-7)
Supplement: Supplementary file 3 [file 12885_2026_17107_MOESM3_ESM.pdf]

A

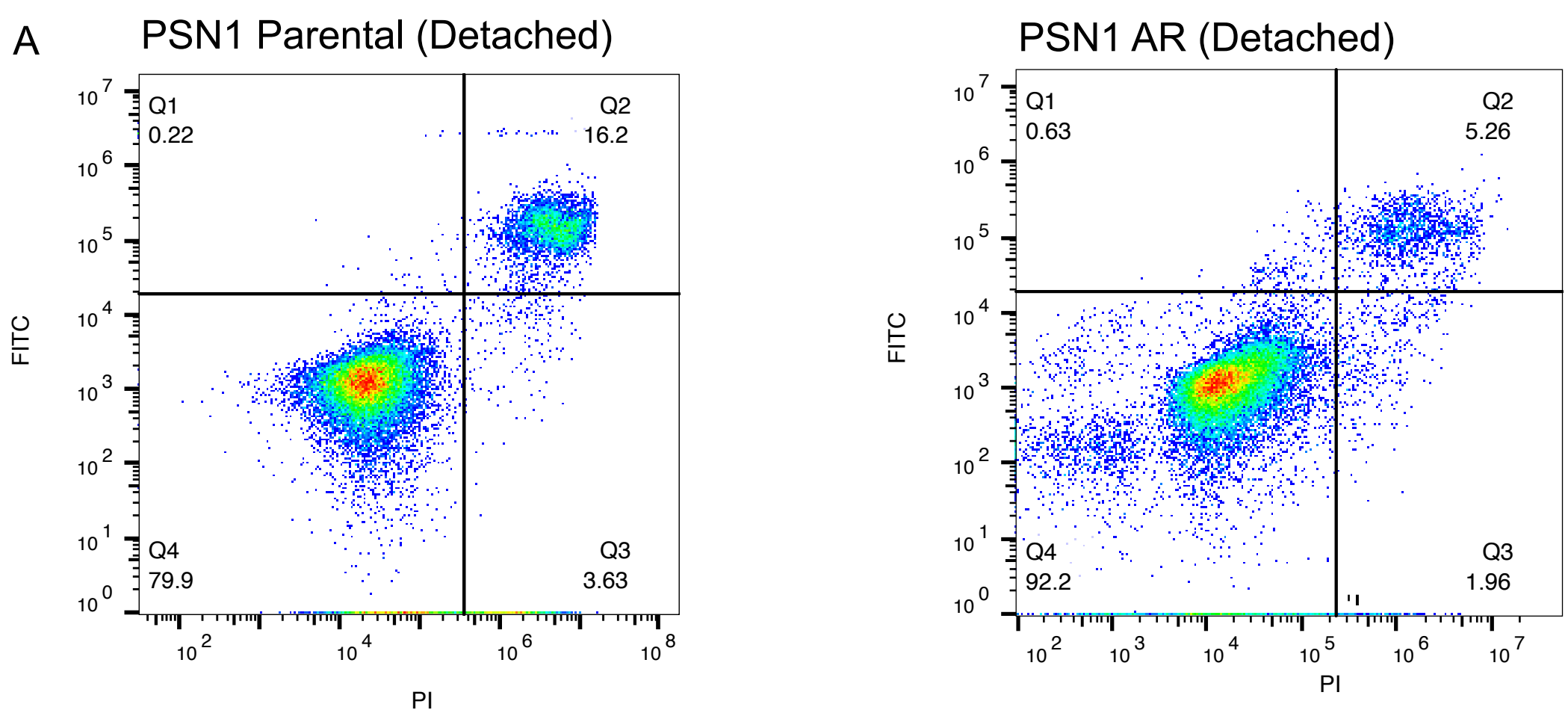
PSN1 Parental (Detached)
Q1
0.22
Q2
16.2
Q3
3.63
Q4
79.9
FITC
PI
PSN1 AR (Detached)
Q1
0.63
Q2
5.26
Q3
1.96
Q4
92.2
FITC
PI

B

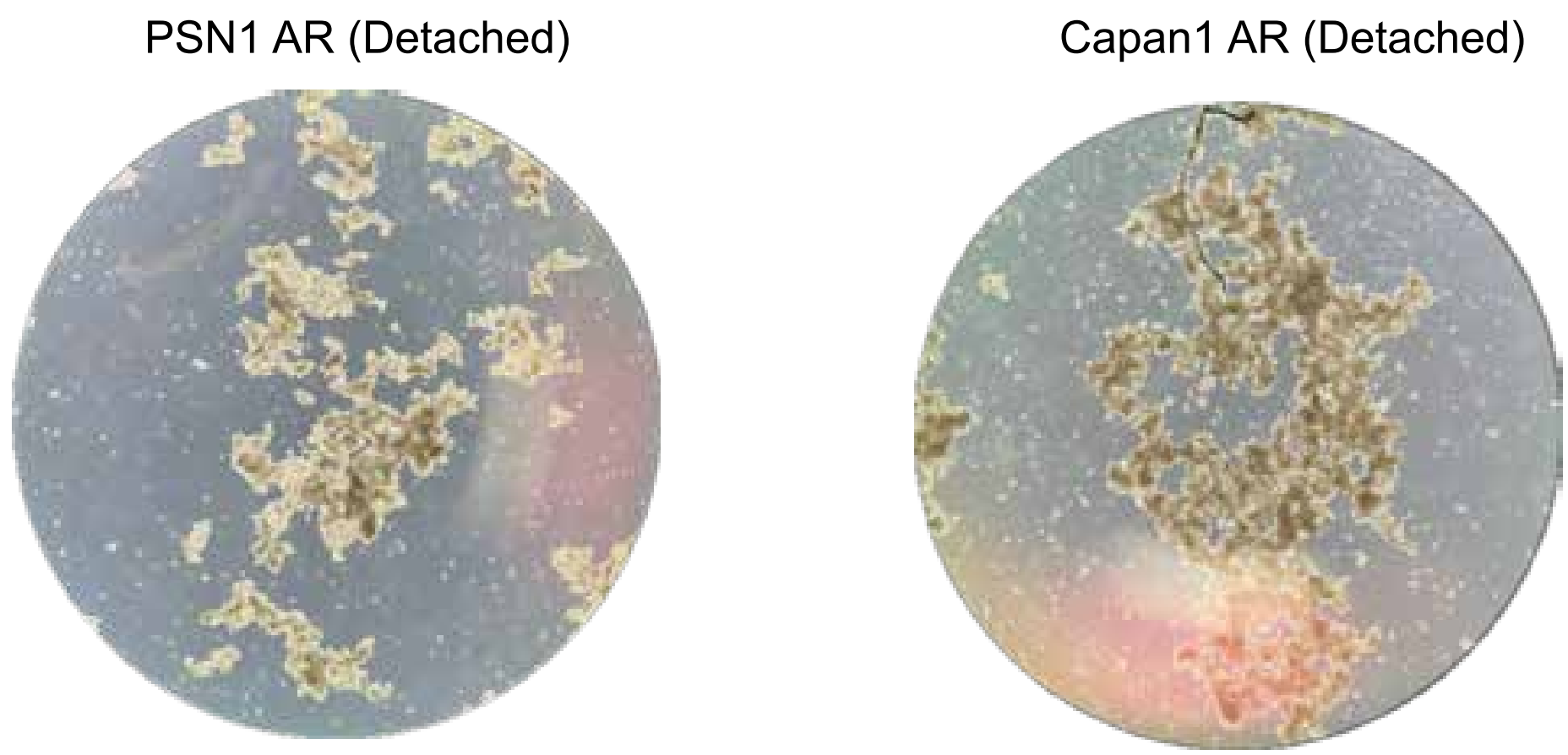
PSN1 AR (Detached)
Capan1 AR (Detached)
